# SPIRITUAL NEEDS OF AGING: BRIDGING RESEARCH AND PRACTICE TO IMPROVE HEALTH OUTCOMES IN OLDER ADULTS

**DOI:** 10.1093/geroni/igad104.1265

**Published:** 2023-12-21

**Authors:** Katherine Britt, Chad Federwitz, Jill Hamilton

## Abstract

Accumulating research reports that spiritual and religious practices are associated with better mental and physical health and support successful aging. Spiritual and religious practices often include the exploration of meaning and purpose in life, connectedness, inner peace, belonging, contentment, and near-end-of-life completion, all of which are tied back to health and health outcomes. However, these experiences and related positive outcomes may not always be meaningfully explored or considered. Social distancing and isolation studies have emphasized the importance of supporting the spiritual needs of older adults for connectedness and meaning. Less is known about how to support these spiritual needs in practice and how to provide spiritual care. This symposium will explore the current status of practices, research, and tools that address the spiritual needs of older adults, particularly in connection to health concerns and quality of life. A focus on current practices towards spiritual care for older adults and future implications regarding research and practice will also be considered. Recommendations for future gerontological inquiry into the importance of spiritual care and diverse approaches within gerontological practice will be highlighted and discussed. Dr. Zhao will describe the spiritual needs of persons living with dementia (PLWD). Dr. Epps will present the effectiveness of a culturally relevant and faith-based home toolbox for Black caregivers of PLWD. Dr. Lekhak will highlight the role of compassionate love in mediating the relationship between meditation practice and mental health. Dr. Britt will present religious attendance associations with better sleep quality in older adults to support cognitive function. This is a Religion, Spirituality and Aging Interest Group Sponsored Symposium.

